# Frailty and suicidal ideation among older adults living alone in the community: a moderated mediation model of perceived burdensomeness and positive emotions

**DOI:** 10.3389/fpubh.2024.1392424

**Published:** 2024-10-30

**Authors:** Yang Yang, Xinyue Zhang, Dan Zhang, Yonggang Su

**Affiliations:** ^1^Journal Center of Shandong First Medical University and Shandong Academy of Medical Sciences, Jinan, China; ^2^College of Radiology, Shandong First Medical University and Shandong Academy of Medical Sciences, Jinan, China; ^3^Department of Nursing, The First Affiliated Hospital of Shandong First Medical University and Shandong Provincial Qianfoshan Hospital, Jinan, China; ^4^Department of Medical Psychology and Ethics, School of Basic Medical Sciences, Shandong University, Jinan, China; ^5^School of Nursing and Rehabilitation, Shandong University, Jinan, China; ^6^School of Foreign Languages and Literature, Shandong University, Jinan, China

**Keywords:** frailty, suicidal ideation, perceived burdensomeness, positive emotions, older adults living alone in the community, moderated mediation model

## Abstract

**Background:**

Suicide rates among older adults individuals living alone in the community are comparatively high. The prevalence of suicidal ideation among older adults living alone in the community was assessed using the interpersonal-psychological theory of suicide and the broaden-and-build theory of positive affect.

**Objectives:**

The research objectives of this study was examine to the prevalence of suicidal ideation among older adults living alone in the community. It was examined whether perceived burdensomeness mediated the relation between frailty and suicidal ideation, and whether positive emotions moderated either the indirect or direct effect of a mediation model.

**Methods:**

The model was tested on 893 older adults people living alone in the community in Xintai City, China. An assessment of participants’ frailty, suicidal ideation, perceived burdensomeness, and positive emotions was conducted.

**Results:**

The results demonstrated that perceived burdensomeness acted as a partly mediating factor in the relationship between frailty and suicidal ideation. In addition, the direct and indirect effects of the mediation model were moderated by positive emotions. When levels of positive emotion were high, fragility had a weaker effect on suicidal ideation, and perceived burdensomeness had a weaker effect on suicidal ideation.

**Conclusion:**

Results emphasize that interventions aimed at improving positive emotions could have a protective effect on frail older people living alone in the community who are at risk of suicide.

## 1 Introduction

Increasing population ageing is undoubtedly a huge challenge for both developed and developing countries (1). In the upcoming 25 years, China will become a “super senior society,” with 24.71% of the geriatric population (aged 65 and over), and a significant increase in the proportion of older adults living alone (2). Influenced by traditional Chinese culture, co-residence between the older adults and their children is a core component of the Chinese family aging model. However, with the increasingly obvious trend of family nucleation, the weakening of traditional family values as well as the fading of filial piety culture, the residence pattern of the older adults has also changed significantly, and the number and proportion of older adults living alone is rapidly increasing. Additionally, the proportion of older adults persons (65 years of age or older) who live alone grew from 6.1% in 2010 to 7.5% in 2019 and is expected to continue rising (3).

Unfortunately, a number of medical, psychological, and social issues are linked to older persons who live alone (4), such as feeling isolated (5), experience loss of function (6), socioeconomic disadvantage (7), and even a higher risk of suicide. Barraclough discovered that the prevalence of suicide among older adults was more strongly associated with living alone than with any other social variable (8). Other study had concluded that from 19 to 60% of older suicides lived alone at the time of death (9). Hu et al. (10) found the prevalence of suicidal ideation among older adults living alone to be 23.6%, much higher than the results of a meta-analysis of suicidal ideation among Chinese older adults by Dong et al. (11) which was 14.7%. Additionally, there is evidence indicating that a greater proportion of suicide completers reside alone as opposed to with their families (12).

The novel coronavirus infection (COVID-19) (13) has resulted in a reduction in social interactions and isolation due to its high transmissibility (14). The pandemic and associated physical distance measures weaken or even limit the possibilities for intimacy and affection with loved ones or friends (15), and the resulting reduction in social contact outside the home may result in the marginalization of older adults who live alone. During the pandemic, it is postulated that older adults who live alone may be at an increased risk of adverse health outcomes in comparison to those who live with others. This is attributed to a lack of direct support and access to basic health care, in addition to the reduced availability of routine services (16). It is probable that these factors will contribute to an elevated risk of suicide among older adults who reside alone. Given that suicidal ideation is the most significant predictor of suicide attempts and mortality in older adults, it is particularly important to address suicidal ideation among older adults living alone in China, especially in the context of COVID-19 pandemic.

Living alone usually leads to a variety of adverse physical and psychological changes that ultimately affect healthy aging and quality of life in older adults, one of the major adverse features of which is frailty (17). The term “frailty” has been employed in clinical contexts to describe a condition that commonly affects older individuals and is associated with impaired strength, endurance, and balance, along with heightened vulnerability to trauma and other forms of stress. It is also associated with an elevated risk for morbidity, disability, and mortality. The prevalence of frailty among individuals aged 65 and older is estimated to range from 10 to 25%, with the proportion increasing significantly with increasing age (18). The condition of living alone is frequently included as one of the criteria in a number of tools designed to assess social frailty or social aspects of frailty (19), and a study demonstrated that older adults with social frailty exhibited a heightened risk of developing physical frailty (20). The findings of a systematic review and meta-analysis indicated that older adults residing in their own homes in the community are significantly more prone to frailty in comparison to those who reside in other living arrangements (21). It has been demonstrated that frailty represents a significant risk factor for suicidal ideation among older adults (22). And some evidence has suggested frailty characteristics in older adults may lead to hastening the spiral of suicidal ideation (23). However, the underlying mechanism of the relationship between frailty and suicidal ideation has not yet been exhibited. Nevertheless, the underlying mechanism by which frailty is associated with suicidal ideation in older adults living alone remains unclear.

According to the development model of suicide trajectories, frailty as a somatic stressor (distal factor), its association with suicide may be mediated by further psychological stressors (proximal risk factors) (24). Previous studies found that the frailty in older adults with chronic diseases can lead to perceived burdensomeness, then developed into depression, frustration, etc., and even suicidal behavior (25), suggesting the potential effect of perceived burdensomeness in mediating physical risk factors and suicidal ideation. Perceived burdensomeness (PB) have been proposed by the Interpersonal-Psychological Theory of Suicide (26) as a proximal adequacy contributor to suicide ideation, especially in older adults. PB is a distorted perceptions that one is so incapable that one is a burden or liability to other people, and it became stronger with aging (26). These misconceptions can lead to shame, low self-esteem, and self-hatred, which can cause individuals to believe that their death is more valuable to those around them than their own survival (27). A previous study found that impairment in self-care and social functioning may be more strongly associated with perceived burden (28). Since older adults who live alone face more social isolations and self-care challenges, and frailty may impair their self-care functions, which can bring a greater sense of perceived burden. Therefore, it is reasonable to hypothesize that the association of frailty and suicidal ideation may be mediated by perceived burdensome in older adults living alone. However, the existence of positive buffering factors that may play a role in preventing the development of negative outcomes is suggested by the fact that not all frail older adults perceive distress and exhibit suicidal ideation. To reduce the risk of suicidal ideation, it is important to elucidate the mechanisms by which protective factors operate.

Positive emotions may be one of such protective factors. Positive emotions represent a psychological strength that can confer psychological adjustment, physical health, Fredrickson et al. (29). Based on the broaden and build theory of positive emotions, positive emotions help build psychological resources, support coping and problem-solving processes (30), and promote individuals’ rapid recovery from stress and enhance their ability to bounce back from adversity. Numerous studies found that positive emotions can buffer the relationship between stress-related risk factors and negative outcomes in general population, such as depressive disorder symptoms (31), and non-suicidal self-injury (32). The theory of socio-emotional selectivity proposes that the ability to regulate emotions increases with age as older adults give priority to emotional and relational goals (32). Gross and colleagues also proved that older adults experience fewer negative emotions than younger adults, increasing focus on the experience of positive emotions (33). Thus we hypothesize that the protective effect of positive emotions may be more pronounced in older adults who live alone, and it could moderate the relationship between frailty (somatic stressor), perceived burdensomeness (psychological stressors) and suicidal ideation.

**Figure 1:**
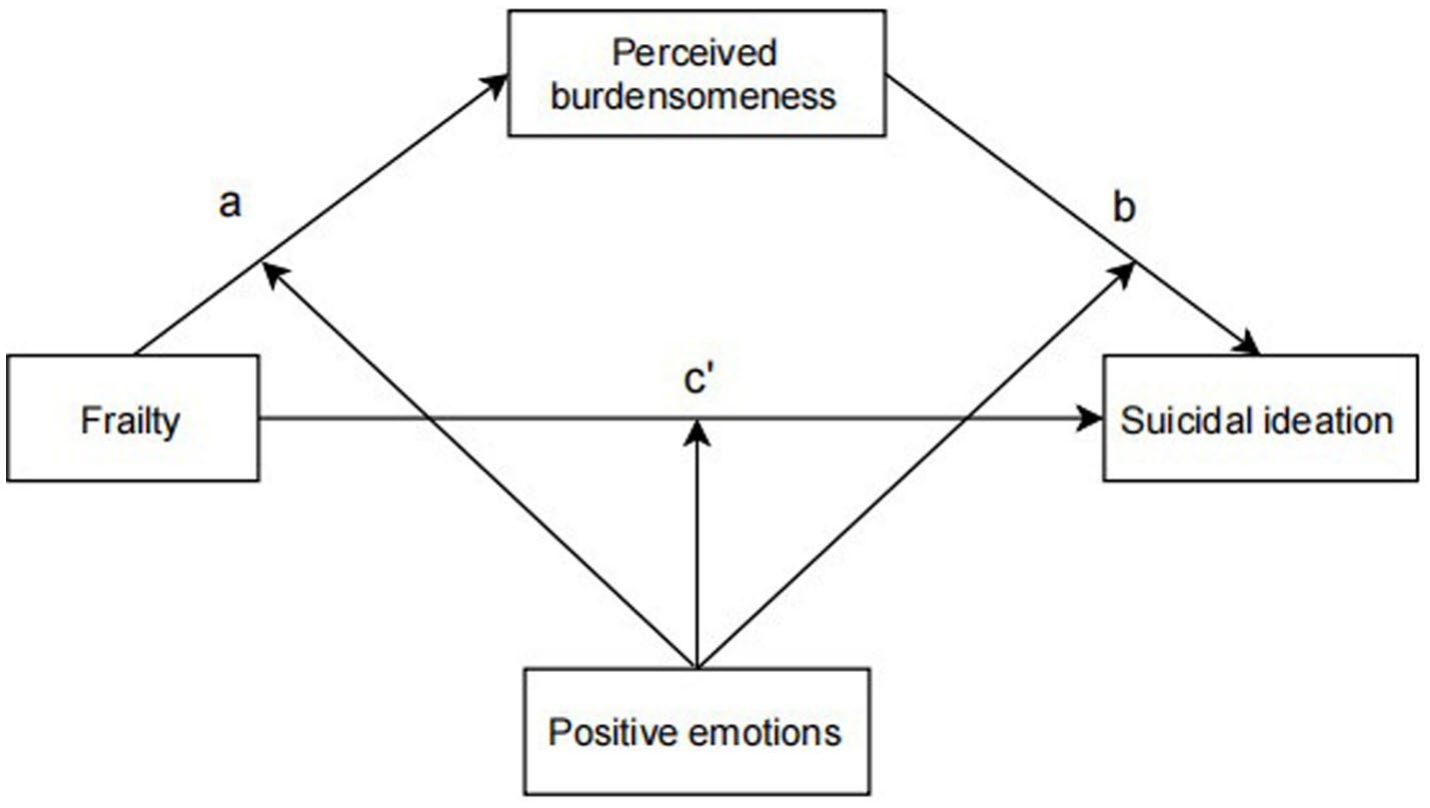
Conceptual model.

Taken together, frailty, perceived burdensome and positive emotions all play an important role in the endorsement of suicidal ideation, but the possible influence of these mechanisms on suicidal ideation among older adults living alone in the community is not clear. The present study aimed at a mediating role model with moderating effects (see Figure 1), which examines the direct relationship between frailty and suicidal ideation as well as the mediating and moderating role of perceived burdensomeness and positive emotions. The hypotheses are as follows: frailty may develop into suicidal ideation either directly (H3) or through the mediating role of perceived burdensomeness (H1, H2), and the hypothetical paths can be moderated by positive emotions (H4, H5, H6).

## 2 Methods and material

### 2.1 Participants

A cluster sampling method was used to collect data in this study from September 2021 to March 2022 in Xintai City, Shandong Province, China. The method of cluster random sampling was adopted in this study,12 of the 22 communities of Xintai City were randomly selected according to the estimated sample size and the number of older adults living alone in each community, and all the older adults in the 12 communities meeting the following criteria were included in this study: (1) aged ≥60 years, (2) living in the target communities during the investigation and for the duration of at least 1 year, (3) capable of verbal communication, and (4) willingness to participate and signing of a declaration of agreement. The exclusion criteria were: (1) severe hearing impairment that could impair communication (with others), (2) a diagnosis of “dementia” or severe cognitive difficulty as indicated by a Mini-Mental State Examination (MMSE) score ≤ 9 (34), (3) receiving a psychotherapy in the past 3 months, (4) with an active or terminal illness.

Interviews were completed in the resident’s apartment or community senior centers, and lasted approximately 1 h. Investigators had to check the quality of the questionnaires and collect them after the interview.

*A priori* analysis was conducted with the equation (*N* = U*α*2 P (1-P)/d2) to determine the required sample size. According to the existing literature, the prevalence of suicide ideation among the Chinese community older adults is about 23.6% (35), thus we assumed the P to be 0.167. We assumed the significance level (α) to be 0.05, the allowed level of error (d) to be 1/5. The results indicated that a minimum number of 311 participants would produce the desired power. Considering the 20% failure/attrition rate, the sample size should not be less than 389 people. In addition, one of the central hypotheses of this study is the mediating role of perceived burdensomeness in the relationship between frailty and suicidal ideation. Referring to the “MedPower” procedure recommended by Kenny for the estimation of sample size and test efficacy of the mediating effect, assuming that the standardized set path coefficients (Beta) of the mediating effect’s first segment (frailty→perceived burdensomeness) and its second segment (perceived burdensomeness →suicidal ideation) are both 0.2, then the mediating effect is 0.04 at the standardized path coefficient (Beta) of the direct effect’s path (frailty→suicidal ideation). Assuming that the Standardised Path Coefficients (Beta) for both the pre (frailty → perceived burdensomeness) and post (perceived burdensomeness → suicidal ideation) mediating effect paths and the direct effect path (frailty → suicidal ideation) are both 0.2, the mediating effect size would be 0.2*0.2 = 0.04, at which point at *α* = 0.05, with a desired efficacy value (power = 0.8), the expected sample size would be 250 individuals. Considering the 20 per percentcent dropout rate, the sample size should be no less than 313. In this study, a total of 968 older adults who met the inclusion criteria were contacted, with 925 agreeing to participate and 43 declining to participate. Of those who agreed, 18 failed to complete the questionnaire, and 14 were excluded because their missing data was more than 15%. Finally, 893 older adults were enrolled, with a response rate of 92.3%.

### 2.2 Research objectives

The primary research aims were to explore the relationship between vulnerability and suicidal ideation in older people living alone and to explore the mediating moderating role of perceived sense of burden and positive emotions. The secondary research objectives were to explore the current status of frailty and the current status of suicidal ideation in older patients living alone.

### 2.3 Variables and measures

#### 2.3.1 Frailty

This study used the Chinese version of Edmonton Frail Scale (EFS) to assess the frailty of subjects, which includes nine dimensions: cognition, health status, independent living, social support, medication, nutritional status, mood, urinary incontinence and mobility, with 11 items. The total score of the scale was 0–17. Finally, according to the needs of the study, the frailty was divided into non-frailty group (≤5) and frailty group (>5) according to dichotomous classification, and into non-frailty group (≤5), mild frailty group (6-7), moderate frailty group (8-9) and severe frailty group (≥10) according to multiple classification (36). The scale has been shown to have high levels of validity and reliability among Chinese older adults living in community (37). The Cronbach coefficient for this study was 0.753.

#### 2.3.2 Perceived burdensomeness

The Interpersonal Needs Questionnaire (INQ)-burdensomeness subscale was used to assess participants’ perceived burdensomeness, INQ is a 15-item scale developed by Van Orden et al. (38) based on the interpersonal theory of suicide. The scale had good validity and reliability among Chinese (39). The Cronbach coefficient for this study was 0.921.

#### 2.3.3 Positive emotions

The Positive and Negative Affect Scale (PANAS) was used to measure participants’ positive emotions. PANAS was developed by Watson et al. in 1988 to assess respondents’ emotions in the last 1–2 weeks, and is the most widely used scale for measuring emotions (40). The English version was translated into Chinese by Huang Li et al. in 2003 (41). The scale consists of 10 items each of positive and negative emotions, each of which is scored from 1 to 5, representing “almost none,” “less,” “moderate,” “more,” and “more,” Each item is scored from 1 to 5, representing “almost none,” “less,” “moderate,” “more,” and “extremely.” The Cronbach coefficient for this study was 0.919.

#### 2.3.4 Suicidal ideation (SI)

Participants’ suicidal ideation in the past week was assessed using the Beck Suicidal Ideation Chinese Version Inventory (BSI-CV). The scale consists of 19 items, with the first 5 items being screening items. Questions 6–19 should only be asked if the answer to item 4 (active suicidal thoughts) and item 5 (passive suicidal thoughts) is yes. Each item was given a score ranging from 0 to 2, and the total score ranged from 0 to 38. The higher the score, the stronger the suicidal ideation (42). The scale has been shown to have a high degree of validity and reliability among older adults in China (43). The Cronbach coefficient for this study was 0.942.

#### 2.3.5 Sociodemographic covariates

Sociodemographic covariates included age, gender, marital status, education, self-rated financial status, family visit frequency, and history of attempted suicide.

#### 2.3.6 Physical and mental health covariates

Physical and mental health covariates included comorbidities, depression, and cognitive function, as measured by the Medical Disorders (MD) scale, PHQ-9, and MMSE.

##### 2.3.6.1 Comorbidities

The quantity of chronic diseases was evaluated using the Chronic Disease Quantity Questionnaire (44), which lists twelve common chronic diseases in the older adults: diabetes, hypertension, osteoarthritis, liver disease, kidney disease, cancer, congestive heart failure, chronic obstructive pulmonary disease, heart attack, gastrointestinal disease, hearing impairment, and eye disease, and requires the older adults to answer whether they have one or more of these diseases, and records the number of diseases in the older adults.

##### 2.3.6.2 Depression

The Patient Health Questionnaire (PHQ-9) served to measure symptoms of depression. There were nine items, and each item was scored from 0 to 3, and the total score was 27. The higher the score, the more severe the depression symptoms. The scale is widely available and proven to show great validity and reliability among older adults (45). The Cronbach coefficient for this study was 0.946.

##### 2.3.6.3 Cognitive function

Cognitive functioning was evaluted using a Mini-Mental State Examination (MMSE), with 30 terms and five aspects including orientation, registration, attention and calculation, memory and language ability. One point is given for each correct answer. Overall score ranges from 0 to 30, with higher scores representing greater cognition function, scores ≤24 indicating impaired cognitive function in older adults, and scores≤9 indicating severely impaired cognitive function (46). The scale is widely available and proven to show great validity and reliability among older adults (47). The Cronbach coefficient for this study was 0.789.

### 2.4 Statistical analysis

Descriptive statistics, independent samples t-tests, chi-squared tests, and one-way analysis of variance (ANOVA) were used to describe the sociodemographic characteristics and to compare the distribution of suicidal ideation, respectively. Pearson correlation analyses were used to reflect correlations between the core variables (frailty, perceived burdensomeness, suicidal ideation, and positive emotions). Next, the PROCESS 4.0 macro program plug-in developed by Hayes et al. was used to conduct the mediation model and moderated mediation model analyses (48). The bias-corrected 95% confidence interval (CI) was calculated using 5,000 bootstrapping resamples. Model 4 was used to examine whether the association between frailty and suicidal ideation was mediated by perceived burdensomeness and Model 59 explored the moderated mediation effect, which is whether positive emotions moderated the direct and indirect effects of frailty on suicidal ideation (49). In addition, all models were controlled for covariates (gender, self-rated financial status, children visit frequency, history of attempted suicide, the total number of chronic illnesses, depression, and cognitive function) and the study variables were standardized. In addition, the simple slopes graphs was also shown by using the interactive utility tool (50). All analysis was carried out with SPSS 26.0 software (IBM, Armonk, NY, USA) and R 4.2.0, and a two-tailed *p*-value of < 0.05 was defined as being statistically significant.

## 3 Results

### 3.1 Basic characteristics

The sociodemographic characteristics were shown in Table 1. A total of 893 older adults living alone in the community participated in the study. There were 435(48.7%) females and 458 (51.3%) males. The mean age was 74.98 (SD = 6.71), with an age range of 64 to 90 years old. The mean of time spent living alone was 5.85(SD = 3.09), the mean of number of chronic diseases was 1.92(SD = 1.54), and the mean of cognitive function was 26.38(SD = 3.89), most older adults living alone in the community reported primary school education or under (56.4%), a fair financial status (52.4%), being widowed (62.8%), and been visited by children once per 1–3 month (51.8%) and Table 1 showed the univariate analysis of suicidal ideation for all respondents in demographic factors. By comparison using independent-samples t-test and chi-square tests, the differences were found to group respondents’ gender, the number of chronic diseases, cognitive function, self-rated financial status, children visit frequency, history of attempted suicide, and depression.

**Table 1:** Sociodemographic characteristics and the distribution of suicidal ideation (*n* = 893).

Variables	Total	NSI (*n* = 754)	SI (*n* = 139)	**χ** ^**2**^ **/t**	*P*
Gender				11.925	0.001
Female	435(48.7%)	386(51.2%)	49(35.3%)		
Male	458(51.3%)	368(48.8%)	90(64.7%)		
Marital status				3.418	0.332
Unmarried	105(11.8%)	86(11.4%)	19(13.7%)		
Married	79(8.8%)	72(9.5%)	7(5.0%)		
Divorced	148(16.6%)	123(16.3%)	25(18.0)%		
Widowed	561(62.8%)	473(62.7%)	88(63.3%)		
Age(mean ± SD)	(74.98 ± 6.71)	74.84 ± 6.78	75.74 ± 6.30	2.400	0.144
Education				0.651	0.885
Illiterate	296(33.1%)	246(32.6%)	50(36.0%)		
Primary school	208(23.3%)	176(23.3%)	32(23.0%)		
Junior high school	213(23.9%)	182(24.1%)	31(22.3%)		
Senior high/above	176(19.7%)	150(19.9%)	26(18.7%)		
Time spent living alone (mean ± SD)	(5.85 ± 3.09)	5.82 ± 0.11	6.02 ± 0.26	4.404	0.479
Number of chronic diseases (mean ± SD)	(1.92 ± 1.54)	1.81 ± 1.44	2.53 ± 1.87	35.504	<0.001
Cognitive function (mean ± SD)	(26.38 ± 3.89)	27.13 ± 3.10	22.30 ± 5.08	117.325	<0.001
Self-rated financial status				12.553	0.002
Poor	239(26.8%)	186(24.7%)	53(38.1%)		
Medium	468(52.4%)	401(53.2%)	67(48.2%)		
Good	186(20.8%)	167(22.1%)	19(13.7%)		
Children visit frequency				41.705	<0.001
Once-four times per month	98(11.0%)	85(11.3%)	13(9.4%)		
Once per 1–3 months	463(51.8%)	422(56.0%)	41(29.5%)		
Once per over 3 months	332(37.2%)	247(32.8%)	85(61.2%)		
History of attempted suicides				14.709	<0.001
No	880(98.5%)	748(99.2%)	132(95.0%)		
Yes	13(1.5%)	6(0.8%)	7(5.0%)		

Note: NSI = participants without suicidal ideation, SI = participants with suicidal ideation.

### 3.2 Bivariate analyses

Table 2 showed the means, SD, and correlations of the main variables. The results showed that suicidal ideation was positively related to frailty (*r* = 0.490, *p* < 0.01) and perceived burdensomeness (*r* = 0.444, *p* < 0.01), and negatively related to positive emotions (*r* = −0.455, *p* < 0.01). Besides, positive emotions were negatively associated with frailty (*r* = −0.265, *p* < 0.01) and perceived burdensomeness (*r* = −0.251, *p* < 0.01). In addition, frailty was positively associated with perceived burdensomeness (*r* = 0.536, *p* < 0.01).

**Table 2:** Bivariate correlation among frailty, suicidal ideation, perceived burdensomeness and positive emotions (*n* = 893).

Variables	Mean	SD	1	2	3	4	5
Frailty	7.92	4.047	1				
Perceived burdensomeness	22.09	9.754	0.536^**^	1			
Depression	10.58	7.899	0.546^**^	0.392^**^	1		
Positive emotions	29.13	7.992	−0.265^**^	−0.251^**^	−0.143^**^	1	
Suicidal ideation	4.75	7.592	0.490^**^	0.444^**^	0.431^**^	−0.455^**^	1

Controlling for gender, self-rated financial status, children visit frequency, history of attempted suicide, the total number of chronic illnesses, depression, and cognitive function. **p < 0.01.

### 3.3 Mediation analyses

As shown in Table 3, mediation results indicated that the overall effect (path C) of frailty on suicide ideation was significant. (*B* = 0.295, *p* < 0.001). The significant coefficient of path a (*B* = 0.444, *p* < 0.001) and path b (*B* = 0.200, *p* < 0.001) indicated positive associations of frailty on perceived burdensomeness and perceived burdensomeness on suicidal ideation. Besides, the indirect effect of frailty on suicidal ideation (path a * b) was statistically significant [*B* = 0.089, 95%CI = 0.016, 0.121]. In addition, the direct effect of frailty on suicidal ideation (path c’ = 0.206, *p* < 0.001) was also significant, indicating that perceived burdensomeness partially mediated the relationship between frailty and suicidal ideation.

**Table 3:** Mediation analysis (*n* = 893).

Variable	Path c		Path c’ and b		Path a		Path a*b			
	B	SE	B	SE	B	SE	B	SE	LLCI	ULCI
Frailty	0.295***	0.323	0.206***	0.035	–	–	0.089	0.016	0.058	0.121
Perceived burdensomeness	–	–	0.200***	0.031	0.444***	0.034				
*R* ^2^_adj_	0.376		0.404		0.307					
F	66.578		66.445		48.931					

Controlling for gender, self-rated financial status, children visit frequency, history of attempted suicide, the total number of chronic illnesses, depression, and cognitive function. ***p < 0.001.

### 3.4 Moderated mediation analyses

**Figure 2:**
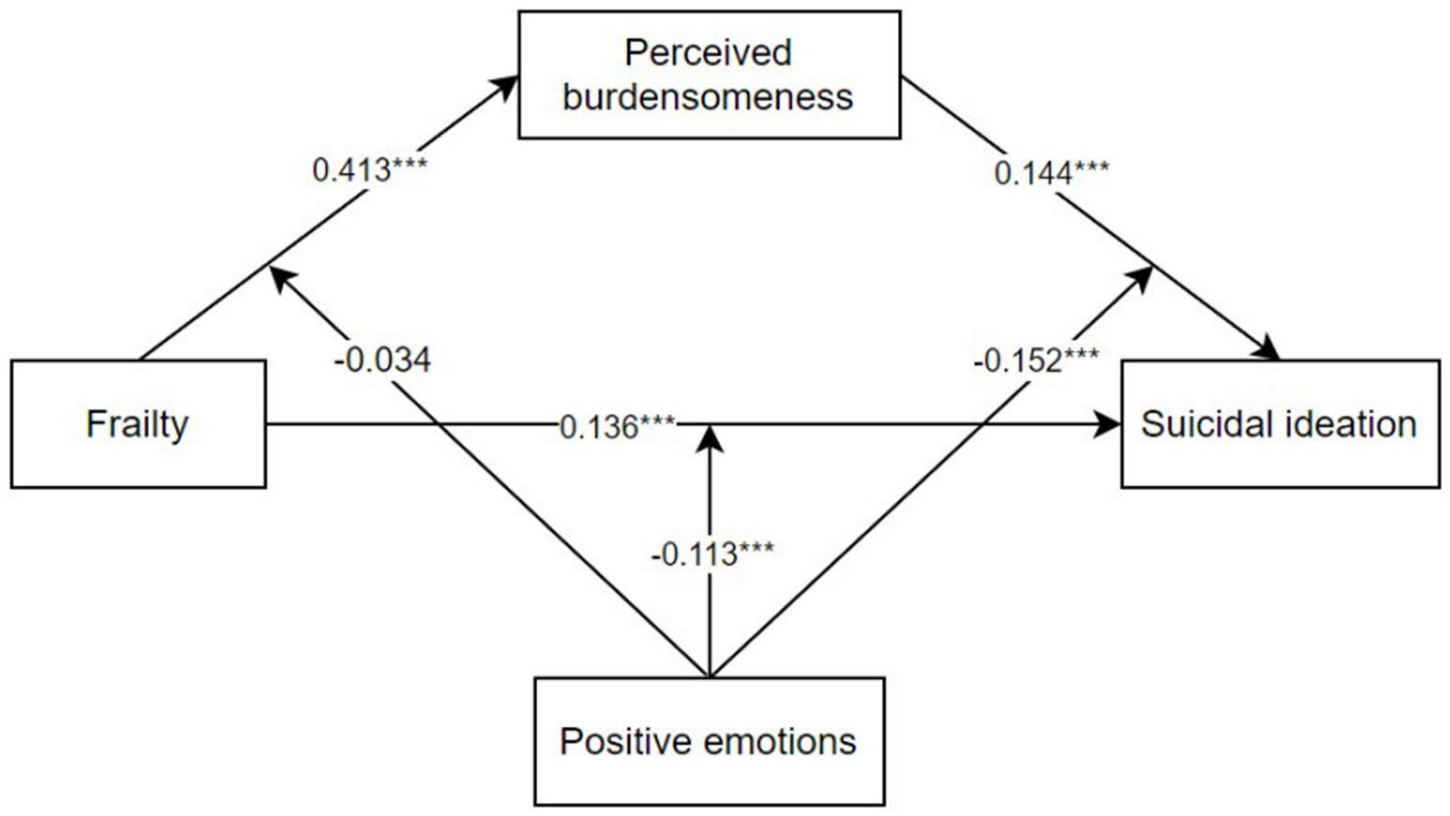
The final moderated mediation model.

Table 4 shows the outcomes of the moderated mediation analysis. The results showed that positive emotions moderated the direct effect (frailty-suicidal ideation) [*B* = −0.113, 95% CI: −0.160, −0.066], and the indirect effect of frailty on suicidal ideation through perceived burdensomeness-suicidal ideation [B = −0.152, 95% CI: −0.202, −0.102]. However, positive emotions did not play a moderating role in the path a (frailty-perceived burdensomeness) of the mediation model [B = −0.034, 95% CI: −0.082, −0.013]. The final moderating model is shown in Figure 2.

**Table 4:** Moderated mediation analysis (*n* = 893).

Variable	B	SE	t	LLCI	ULCI
Outcome: perceived burdensomeness					
Frailty	0.413	0.035	11.915***	0.345	0.481
Positive emotions	−0.108	0.029	−3.690**	−0.165	−0.05
Frailty × Positive emotions	−0.034	0.024	−1.415	−0.082	0.013
Outcome: suicidal ideation					
Frailty	0.136	0.030	4.472***	0.076	0.196
Perceived burdensomeness	0.144	0.027	5.260***	0.090	0.198
Positive emotions	−0.275	0.024	−11.457***	−0.323	−0.228
Frailty × Positive emotions	−0.113	0.024	−4.679***	−0.160	−0.066
Perceived burdensomeness × Positive emotions	−0.152	0.026	−5.931***	−0.202	−0.102

Controlling for gender, self-rated financial status, children visit frequency, history of attempted suicide, the total number of chronic illnesses, depression, and cognitive function. **p<0.01, ***p<0.001.

**Figure 3:**
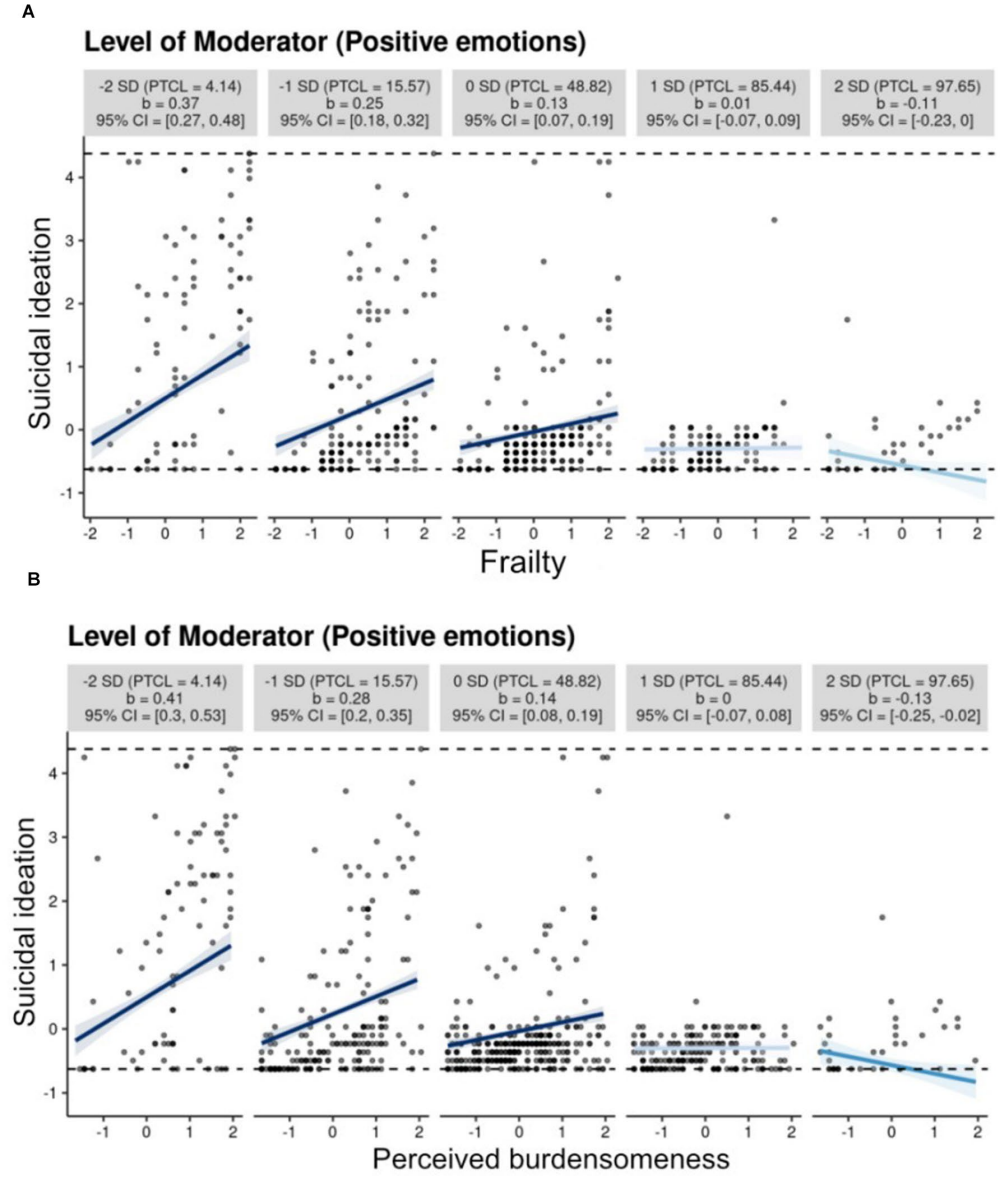
**(A)** Frailty and suicidal ideation, **(B)** Perceived burdensomeness and suicidal ideation.

The conditional effect of frailty on suicidal ideation through burdensome at different levels of positive affect was analyzed to further test the moderating effect. As shown in Figure 3, positive emotions is divided into very low (the mean minus two SD), low (the mean minus one SD), medium (the mean), high (the mean plus one SD), and very high (the mean plus two SD). Figure 3a depicts the effect of frailty on suicidal ideation has been moderated by different levels of positive emotions, and specifically, a higher level of positive emotions predicted a weaker effect of frailty on suicidal ideation. Similarly, Figure 3b depicts that a higher level of positive emotions predicted a weaker effect of burdensome on suicidal ideation.

## 4 Discussion

The negative impact of frailty on suicidal ideation is increasingly supported by empirical evidence. However, the mechanisms underlying this association remain largely unexplored, especially among community-dwelling older adults. The present study found that frailty was significantly and positively associated with suicidal ideation and perceived burdensomeness could mediate the relationship. Furthermore, positive emotions significantly moderated the mediating role of perceived burdensomeness. These results support our hypothesis.

There is a limited amount of knowledge about the prevalence of suicidal ideation among older adults who live alone. In the present study, the prevalence of suicidal ideation among the older adults living alone in the community was 15.57% (139/893), which was significantly higher than the prevalence of community-dwelling older adults in Beijing (2.3%) (51), and that reported in a recent meta-analysis for general Chinese older adults[11.5% (range 2.2 to 21.5%)] (11). The prevalence of suicidal ideation is also higher in this study than in the Korean National Health and Nutrition Examination Survey [10.0% (123/1234)] for people aged 65 years and older (52). Thus, more attention should be paid to the prevalence of suicidal ideation among Chinese older adults living alone, which may be higher.

The prevalence of frailty among the older adults living alone was 50.73% in the study, higher than that of a study on the frailty of older adults people living alone in the Japanese community (male: 38.1%; female: 16.8%) (53). Consistent with findings from previous studies in a population of U.S. veterans aged 65 years or older (54) and older adults living in the community with major depressive disorder (55), the present study also found that frailty is a risk factor for suicidal ideation among Chinese older adults who live alone. Frailty reduces the ability of self-care and participating in social activities, thus may exacerbates the social isolation and loneliness that result from living alone, placing individuals at high risk for suicide.

Previous studies have shown that perceived burdensomeness is a strong and solid predictor in the development of suicidal ideation (55), especially in older populations, over risk variables such as depression (56). The present study confirmed that, i.e., perceived burdensomeness partially mediates the association between frailty and suicidal ideation, suggesting the importance of perceived burdensomeness in predicting suicidal ideation in frail older adults living alone. Furthermore, the mediating effect of perceived burdensomeness was still significant when controlling for depressive symptoms, suggesting a strong effect of burdensomeness on suicidal ideation. The studies regarding perceived burdensomeness in older adults have primarily included older adults experiencing dysfunction or physical illness (26). Some studies have found that perceived burdensomeness is significantly associated with suicidal ideation among older adults with life-limiting illnesses (57). As the interpersonal theory of suicide suggests, many older adults are more likely to experience feelings of burden as they age (26), and perceived feelings of burden may be particularly relevant to older adults (56). Specifically, many older adults experience a sense of distress as they begin to need more care from family members or friends, and the increased need for assistance from others may derive from a medical problem or loss of a job, resulting in an individual’s need for (and not generating) financial support or physical care. Receiving help may make some older adults feel like a burden to others (58). In such cases, some older persons may perceive themselves as a burden and may consider this state to be steadfast and permanent, leading to the consideration of suicide and death as a solution to the problem of ongoing burden (26). And the relationship between perceived burdensomeness and suicidal ideation caused by social, interpersonal and physical constraints may be a mutually enhancing process of decline in old age, increasing the sense of perceived burdensomeness among the older adults, hastening the spiral of suicidal ideation (54).

Therefore, older persons living alone, especially those with physical limitations such as frailty, deserve more physical care and more comprehensive psychological support. When diagnosing frail older adults, perceived burdensomeness can be evaluated firstly to predict the possibility of suicide risk by clinicians, and may help older adults to avoid the potential stigma of discussing suicide and increase engagement with clinical services (59). In addition, frailty and other medical comorbidities can be a valuable tool for clinicians in the identification and intervention of potential suicidal ideation, which may not normally be a topic of discussion, helping frail older adults at this critical time of mental pain and stress (55).

Additionally, the study found that positive emotions mitigated the impact of frailty on suicidal ideation directly, or through the path of perceived burdensomeness to suicidal ideation. This result was found to agree with a previous research conducted in a rural sexual minority group, which found that high levels of positive emotions weakened the pathway between perceived burdensomeness and suicidal ideation (60). An experimental study conducted in a student population also concluded that frustrated interpersonal needs, especially perceived burdensomeness, deplete the individual’s desire to continue living before suicidal ideation ensues, whereas positive emotions enhance enthusiasm for life and therefore resist suicidal ideation in the face of interpersonal adversity (61). This result can be explained by the broaden-and-build theory of positive affect, which implied that positive emotions can help broaden cognitive attention to potential coping resources, and thus to foster positive psychological adjustment (62). Positive emotions are also a source of resilience, which in turn promotes further positive emotions (63). A number of lines of evidence are in favor of the view that positive emotions and resilience are mutually reinforcing (64). On this basis, resilience may be used as a moderating variable to reduce or eliminate the correlation between risk (such as frailty and perceived burdensomeness) and suicide (65). Therefore, it is necessary to strengthen policy interventions in the areas of positive emotions in older adults, and enhancing positive emotional experiences in older adults is important to promote a sense of meaning in their lives.

There are some limitations to this study. First, the generalizability should be treated cautiously because Sample size was constrained, with participants from only one city in mainland China. Future studies may include older adults living alone from different regions or cultural backgrounds for confirmation of the results of this study. Second, this study depended on self-reported questionnaire data, therefore, recall bias and report bias was possible. Third, the present study was a cross-sectional study and failed to identify a possible causal link between frailty and suicide. Future studies could use a longitudinal study design to explore the relationship between the pair.

In our findings, the multiple moderating effects of positive emotions provide the feasibility of implementing a positive emotion intervention among older adults living alone in the community. Considering that most Chinese community healthcare facilities lack professional psychological counselors, nurses are more accessible to the older adults living alone and can be trained through psychological intervention techniques for them as a complement to psychological care in the daily care of the older adults living alone in the future study. By improving the quality of nurses’ psychological care competencies, it is expected to reduce the influence of risk predictors on suicidal ideation in older individuals living alone in the community. It is also necessary to maintain a sense of normalcy and rejuvenation in order to encourage the positive emotions of older adults to function and to promote “active and productive aging.” In addition, because perceived burdensomeness mediates the relationship between frailty and suicidal ideation, perceived burdensomeness can be assessed for frail older adults to predict the likelihood of suicidal risk in future clinical practice.

## 5 Conclusion

To summarize, our data indicate that frailty was related to suicidal ideation, and perceived burdensomeness practically mediated this association. This result supports the interpersonal-psychological theory of suicide in older adults living alone. In addition, the finding of positive emotions moderated the relationship among frailty, burdensomeness, and suicidal ideation in this study, supports the broaden-and-build theory of positive affect, and contributes to developing tailored suicide prevention strategies by enhancing positive emotions for older adults living alone. In the future, more research in this field may wish to explore positive psychological interventions. (e.g., positive emotions as a target for intervention) to enhance the emotional threshold of older adults living alone in the community and reduce suicidal ideation.

## Data Availability

The raw data supporting the conclusions of this article will be made available by the authors, without undue reservation.
